# Preliminary Validation of a Modified Screening Tool for the Early Detection of Chronic Kidney Disease in a Pilot Sample

**DOI:** 10.3390/healthcare14050589

**Published:** 2026-02-26

**Authors:** Lorna Kwai Ping Suen, Margaret Wai Yan Wong, Mark Cheuk Man Tsang, Bonnie Mee Ling Tam, Albert Wing Nang Leung, Rick Yiu Cho Kwan, Edward Wai Ching Shum, Wilson Kin Chung Leung, Simon Ching Lam

**Affiliations:** 1 School of Nursing, Tung Wah College, Hong Kong SAR, China; 2 School of Arts and Humanities, Tung Wah College, Hong Kong SAR, China; 3 School of Graduate Studies, Lingnan University, Hong Kong SAR, China

**Keywords:** Chronic Kidney Disease (CKD), early detection of disease, mass screening, reproducibility of results, pilot project

## Abstract

**Background:** The early detection of chronic kidney disease (CKD) is critical to preventing progression and reducing associated morbidity. The original SCreening for Occult REnal Disease (SCORED) tool has been widely adopted for CKD screening. However, its length and inclusion of items with limited predictive value affect its practicality in specific settings. This study aimed to validate a modified version of the tool (SCORED-M), which has fewer items and improved predictive performance for the early detection of disease. **Methods:** A cross-sectional pilot project was conducted and the diagnostic performance of the revised tool (SCORED-M) was evaluated using receiver operating characteristic analysis, sensitivity, specificity, positive predictive value, and negative predictive value (NPV). Items were selected or excluded based on their statistical significance, odds ratios, and clinical relevance to CKD risk. The optimal threshold score for mass screening was determined through a comparative analysis. **Results:** A total of 116 eligible participants enrolled in this pilot study. SCORED-M, comprising six items, rather than nine, as in the original version, demonstrated superior screening performance. It achieved a higher area under curve (0.89 vs. 0.79), sensitivity (0.97), and NPV (0.97), indicating its improved capability to identify individuals with CKD and rule out those without the condition. The age-related scoring range was recalibrated from 2 to 4 points to a narrower span of 1–3 points, to moderate the influence of age as a standalone risk factor for CKD. Items with limited predictive contribution, such as ‘*I am a woman*’, ‘*I have a history of heart attack or stroke*’, *and* ‘*I have circulation disease in my legs*’, were removed, while clinically relevant variables like ‘*I am diabetic*’, ‘*I have a history of congestive heart failure or heart failure*’, ‘*I have protein in my urine*’, ‘*I have uncontrolled high blood pressure*’, and ‘*I have a history of renal disease*’ were retained. A threshold score of ≥4 was identified as optimal, balancing sensitivity and specificity while supporting resource-efficient screening and ensuring the reproducibility of results. Conclusions: This pilot study provided preliminary evidence that the SCORED-M tool offers a more concise and accurate approach to CKD/diagnosis. While the findings are promising, validation in larger and more diverse populations is necessary to confirm the generalizability of the model and refine it for broader clinical application in mass screening programmes.

## 1. Introduction

Chronic kidney disease (CKD) is a progressive condition marked by the gradual deterioration of kidney function. It poses a considerable global health challenge, with an estimated prevalence of 10–12%, affecting over 850 million individuals worldwide (Kidney Disease: Improving Global Outcomes) [1,2]. The consequences of CKD extend beyond individual health, placing significant strain on healthcare systems due to its links with increased hospitalization rates, premature mortality, and long-term treatment costs [3].

CKD is categorised into five stages, based primarily on the estimated glomerular filtration rate (eGFR) and the presence of renal damage indicators, such as albuminuria. Initially developed by the KDOQI initiative of the National Kidney Foundation (NKF) and subsequently refined by KDIGO guidelines, this staging framework is a vital tool for evaluating disease progression and informing clinical decisions [2]. Stages 1 and 2 are characterised by normal or slightly reduced eGFR values (≥90 and 60–89 mL/min/1.73 m^2^, respectively), along with evidence of kidney damage. Stage 3 is divided into 3a (eGFR 45–59 mL/min/1.73 m^2^) and 3b (eGFR 30–44 mL/min/1.73 m^2^), indicating moderate functional decline. Stage 4 represents severe impairment (eGFR 15–29 mL/min/1.73 m^2^), while Stage 5, also referred to as end-stage kidney disease, is defined by an eGFR below 15 mL/min/1.73 m^2^ or the need for dialysis or transplantation. This classification is crucial for conducting risk assessment, monitoring disease trajectory, and guiding timely therapeutic interventions [4].

The early identification of CKD is essential because the condition often remains clinically silent until it reaches advanced stages. Timely detection enables earlier intervention, which can slow disease progression and improve patient outcomes [4]. The KDIGO 2024 [4] Clinical Practice Guideline underscores the importance of early diagnosis and risk stratification using measures such as eGFR and albuminuria. The guideline advocates for routine screening in high-risk groups and the use of validated tools to support clinical decision-making and personalised care strategies [2,5].

Despite the availability of several screening instruments for CKD, limitations in predictive accuracy and generalizability persist. One widely used tool is the SCreening for Occult REnal Disease (SCORED) questionnaire (9 items), which has demonstrated high sensitivity (92–95%) and negative predictive value (up to 99%), with moderate specificity in identifying individuals at risk for CKD [6,7]. However, its length and inclusion of items with limited predictive value affect its practicality in specific settings. In response to these gaps, this pilot study aims at validating a modified version of the tool (SCORED-M), which integrates additional clinical and demographic indicators believed to strengthen screening performance. The objectives of this study are twofold: (1) to evaluate whether the SCORED-M tool improves the accuracy of early CKD detection compared to the original version and (2) to identify the optimal threshold (cut-off point) for the revised version.

## 2. Methods

### 2.1. Study Design

This was a cross-sectional, pilot study.

### 2.2. Participants and Setting

Participants were recruited at the Integrated Health Clinic (IHC) of Tung Wah College (TWC), Hong Kong, from September 2022 to December 2024. IHC serves as a hub for healthcare and nursing services, as well as teaching, learning, and research activities at TWC. Its primary services include consultations and therapies in traditional Chinese medicine, physiotherapy treatments, and training in the use of N95 respirators, including fit testing. These services are available to academic staff, students, and the public.

### 2.3. Data Collection

**Figure 1:**
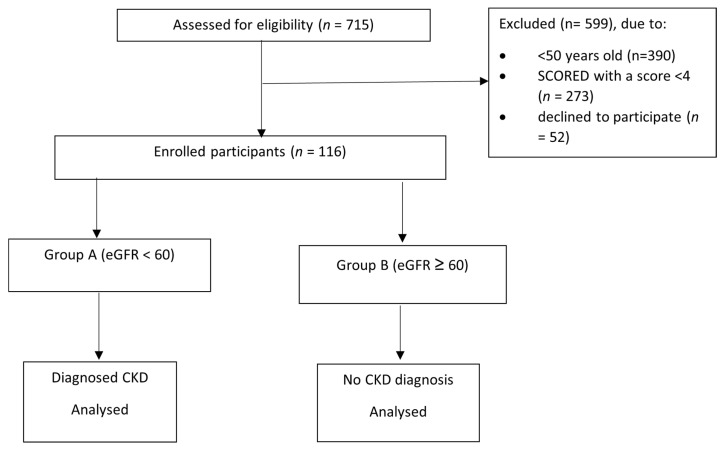
Participant flow diagram for study enrolment. CKD: Chronic kidney disease; eGFR: Estimated glomerular filtration rate; SCORED: SCreening for Occult REnal Disease.

In this study, CKD was defined as an estimated glomerular filtration rate (eGFR) <60 mL/min/1.73 m^2^, calculated from serum creatinine using a standard equation, in line with international guideline definitions of CKD stages 3–5. CKD classification was based on a single eGFR measurement obtained at the time of recruitment; repeat eGFR or albuminuria measurements over ≥3 months, as recommended by KDIGO for the confirmation of chronicity, were not available in this pilot study. Patients who attended IHC were screened for eligibility to participate in this pilot study. Those enrolled met the following criteria: (1) aged 50 years or older; (2) SCORED assessment score of 4 or above, which indicates potential CKD; and (3) provided consent to participate (*n* = 116). A blood test was conducted to measure the eGFR values of the participants. CKD is typically identified when an individual’s eGFR remains below 60 mL/min/1.73 m^2^ for a period exceeding three months, regardless of whether other signs of kidney damage are present. This diagnostic benchmark is broadly recognised in clinical practice, as outlined in the 2021 KDIGO Clinical Practice Guideline for the management of glomerular diseases [8], and is also endorsed by the National Kidney Foundation [9], which highlights eGFR as a key parameter for the early recognition and classification of CKD. The eGFR was calculated using the CKD-EPI equation by inputting the participant’s serum creatinine level, age, and sex into the online calculator provided by the NKF [10]. Participants were divided into two groups according to their eGFR values in the blood test. Those with eGFR <60 mL/min/1.73 m^2^ were assigned to Group A (*n* = 34), while those with eGFR ≥ 60 mL/min/1.73 m^2^ were assigned to Group B (*n* = 82). Those in Group A were diagnosed with CKD, while those in Group B were not. Details of the flow diagram for study enrolment are displayed in Figure 1.

### 2.4. Ethical Consideration

The study was conducted in accordance with the Declaration of Helsinki (1964). Written informed consent was obtained from each participant following a clear explanation of the risks and benefits of the study, provided verbally and in writing. Participation was entirely voluntary, and all prospective participants were informed of their right to decline or withdraw from the study at any point without consequence. Personal data and identifying information were treated with strict confidentiality and anonymity. Ethical approval for the study was sought and granted by the Human Research Ethics Review Committee of TWC.

### 2.5. Tool Revision Process

SCORED is a validated and publicly accessible instrument designed to identify individuals at increased risk of CKD. It comprises nine questions, each corresponding to a clinical or demographic risk factor, including age, hypertension, diabetes, and anaemia. Most items are scored on a binary scale (0 or 1), with age contributing up to four points depending on the respondent’s age group. The cumulative score ranges from 0 to 12, with higher scores indicating a greater probability of impaired kidney function. A commonly accepted cut-off score of 4 or above suggests the need for further clinical assessment. The scoring direction is clinically significant, as it reflects the likelihood of an eGFR falling below 60 mL/min/1.73 m^2^. The SCORED tool has demonstrated strong psychometric characteristics, with validation studies reporting high sensitivity and negative predictive value, supporting its reliability and utility in both community and primary care screening contexts [6,8]. An area under curve (AUC) value of up to 0.88, indicating strong discriminatory power and reliability in CKD detection, has been reported when evaluated against the KEEP guidelines using NHANES and ARIC/CHS datasets [11].

The tool was preliminarily refined (SCORED-rev) following consultations with one of the co-authors (BT), an experienced nurse practitioner specialising in renal care. The revisions were made under the assumption that they would enhance its screening capability for CKD. However, the inclusion of these revisions in the modified version (SCORED-M) in this study was subject to further analyses.

In SCORED-rev, the age variable was revised with a new coding scheme, with 50–59 years old coded as 1, 60–69 years old coded as 2, and 70 years old or above coded as 3. Two items from the original version were modified: ‘*I have high blood pressure*’ was changed to ‘*I have uncontrolled high blood pressure*’, and ‘*I am diabetic*’ was revised to ‘*I have uncontrolled diabetes*’. Two new items believed to be associated with CKD were also introduced: ‘*I have a history of renal disease(s)*’ and ‘*I have a history of urinary problem(s)*’. These amendments resulted in a revised tool comprising 11 items, with a maximum attainable score of 13 (Table 1).

### 2.6. Statistical Analysis

The following sections highlight various statistical approaches to answer our research questions. Statistical software SPSS (version 30.0) was used unless otherwise specified.

- Descriptive statistics were used to present the participants’ characteristics.
- Univariate logistic regression was applied to all nine items of the original SCORED tool and the four newly added/revised items in SCORED-rev to evaluate the individual predictive power of each item for the presence or absence of CKD, which is the binary outcome measured by eGFR <60 mL/min/1.73 m^2^ (single measurement at study entry). This statistical method estimates the odds ratio (OR), *p*-value, and AUC for each item, allowing researchers to determine whether a specific factor is significantly associated with CKD when considered independently. Items demonstrating statistical significance (*p* < 0.10) were chosen for inclusion in the complete model for multivariate analyses. In addition, items from the original tool were prioritised for inclusion, unless multicollinearity exists or a similar item (i.e., age group) exhibited a relatively higher *p*-value or a lower AUC value, in which case they were considered for removal.
- Correlational analyses using the phi coefficient or Kendall’s tau as appropriate were used to understand the relationships between specific items and the presence/absence of CKD or between items. If two highly correlated items exist, then only one might be retained to avoid multicollinearity.
- Backward logistic regression was used for multivariate analyses. To mitigate overfitting risk inherent to small pilot datasets (*n* = 116), pre-specified statistical thresholds (univariate *p* < 0.10; multivariate *p* < 0.05) were employed for item selection, and clinically justified over purely data-driven selection was prioritised. The decision to retain or remove items was primarily based on these pre-specified statistical thresholds, model fit criteria (e.g., Akaike information criterion [AIC]), and/or clinical relevance to CKD risk factors.
- Receiver operating characteristic (ROC) curve assessment and AUC values were calculated for the original SCORED and modified models.
- DeLong’s test was used to compare the difference in AUCs between the original and optimal multivariate models, using R software (version 4.5.1).
- The diagnostic metrics, namely, sensitivity, specificity, positive predictive value (PPV), and negative predictive value (NPV), and Youden’s J statistics were computed at different cut-off points to determine the desirable threshold of the optimal model. This statistic is commonly used to maximise the combined sensitivity and specificity of a diagnostic test [12].
- Post hoc power analysis confirmed >80% power to detect AUC > 0.70 vs. chance at α = 0.05 despite pilot sample size (*n* = 116).

## 3. Results

To ensure transparency and completeness in reporting, this study adheres to the ‘Standards for Reporting Diagnostic Accuracy Studies’ (STARD) 2015 guidelines [13] (Supplementary Table S1).

### 3.1. Participant Characteristics

The 116 participants comprised 74 females (63.8%) and 42 males (36.2%). The mean age was 71.3 (SD = 7.76) and the mean eGFR was 71.4 (SD = 24.49). Participants were recruited from a single primary care centre in IHC, reflecting typical high-risk screening populations but limiting immediate generalisability to community-dwelling or multi-ethnic cohorts.

### 3.2. Tool Performance

#### 3.2.1. Univariate Logistic Regressions and Correlational Analyses

Table 2 shows the results of univariate logistic regression and correlational analyses on the 13 items previously described. The odds ratio, *p*-value, and AUC of each item were assessed to determine their suitability for inclusion in the comprehensive multivariate model. The original age group variable (coded as 2: 50–59, 3: 60–69, and 4: 70 and above) was replaced with a revised coding scheme (1: 50–59, 2: 60–69, and 3: 70 and above), as direct model comparison demonstrated higher OR (1.70 vs. 1.48) and improved age-specific AUC (0.62 vs. 0.61), yielding the final six-item SCORED-M with overall AUC 0.89.

Multicollinearity was observed between the variables ‘*I have high blood pressure*’ and ‘*I have uncontrolled high blood pressure*’, as indicated by a Kendall’s tau coefficient of 0.433 with a statistically significant *p*-value (<0.001), suggesting a moderate positive association. Owing to this overlap, only one variable was retained for further analysis. ‘*I have uncontrolled high blood pressure*’ (an item in SCORED-rev) was selected because it demonstrated stronger predictive performance, with a higher OR (2.95 vs. 2.69), greater AUC (0.63 vs. 0.60), and a more significant *p*-value (0.01) compared to the alternative. This decision supported model parsimony while preserving explanatory power.

Similarly, multicollinearity was identified between the variables ‘*I am diabetic*’ and ‘*I have uncontrolled diabetes*’, as evidenced by a Kendall’s tau coefficient of 0.451 with a statistically significant *p*-value (<0.001), indicating a moderate positive association. Given this overlap, only one variable, ‘*I am diabetic*’ (an item in the original SCORED), was retained for further analysis due to its stronger predictive performance, reflected in a higher OR (1.93 vs. 1.11) and greater AUC (0.58 vs. 0.51) compared to ‘*I have uncontrolled diabetes*’. This choice enhanced model efficiency while preserving statistical robustness (Table 2).

#### 3.2.2. Multivariate Analyses

Multivariate analysis was conducted using backward logistic regression for the original SCORED model and the comprehensive model developed following preliminary evaluations. Decisions regarding the inclusion or exclusion of items in the optimal model were guided primarily by statistical significance (*p*-values), model fit indicators (e.g., AIC), and clinical relevance. Comparative assessments of AIC values, percentage of correctly predicted cases, overall model quality, and AUC were carried out (Table 3), and the multivariate analyses led to the sequential exclusion of the following items from the models: (Q6) ‘*I have a history of heart attack or stroke*’, (Q3) ‘*I have or have had anaemia*’, (Q2) ‘*I am a woman*’, (Q8) ‘*I have circulation disease in my legs*’, and (Q13) ‘*I have a history of urinary problem(s)*’.

In the optimal multivariate model (Model 6), the item ‘*I have a history of urinary problem(s)*’ was excluded despite Model 6 showing a marginally lower AUC (0.89 vs. 0.89) and a slightly higher AIC (100.48 vs. 99.67) than Model 5. Empirical and methodological considerations justified this exclusion: in the univariate logistic regression, the item yielded a weak predictive value (OR = 0.43) and a weak association with CKD (Phi coefficient = −0.17), indicating limited utility in identifying CKD (Table 3). Although the *p*-value for the item ‘*I have a history of congestive heart failure or heart failure*’ was only marginally statistically significant (*p* = 0.059), it was retained in the optimal model due to its notably high OR (22.87). Additional variables included in the optimal model were the self-reported items ‘*I have protein in my urine*’ (*p* < 0.005) and ‘*I have a history of renal disease*’ (*p* < 0.001), both of which showed statistically significant associations with the outcome. The ORs, Wald statistics, and *p*-values for the retained items in the optimal model (i.e., SCORED-M) are presented in Table 4, while the modifications made to individual items along with their respective justifications are outlined in Table 5.

#### 3.2.3. ROC/AUC of SCORED and SCORED-M

**Figure 2:**
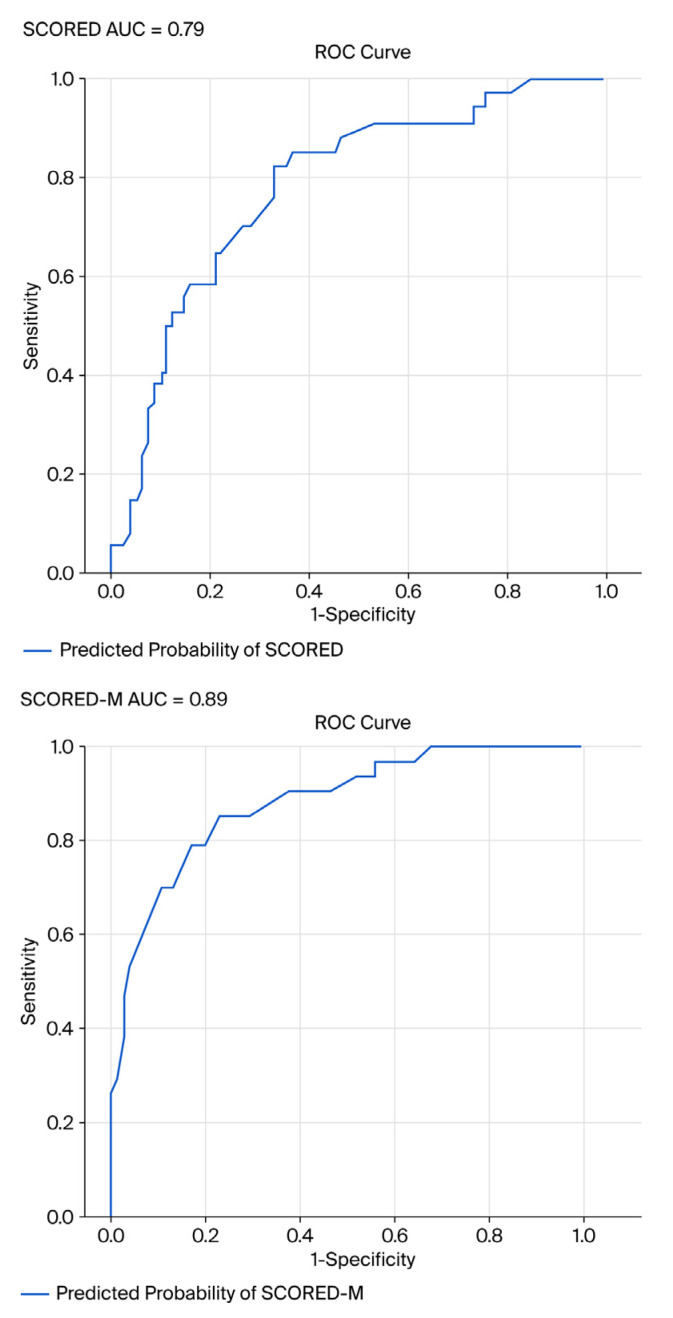
Comparisons of the ROC/AUC of the original SCORED and SCORED-M. AUC = area under the curve.

An evaluation of the ROC curves was carried out for the original SCORED tool and the modified version (SCORED-M). The AUC was used as a key metric to assess the discriminative ability of each model. The findings revealed that SCORED-M demonstrated superior performance, with an AUC of 0.89 compared to 0.79 for the original SCORED in this pilot study. This improvement indicated that the revised model offers enhanced accuracy in distinguishing between individuals with and without the condition of interest, thereby supporting its suitability for CKD screening (Figure 2). In addition, DeLong’s test revealed a statistically significant difference in the AUC between the two models (z = −2.45*; p* = 0.014), indicating that SCORED-M demonstrated a superior discriminative ability compared to SCORED (Table 6). Given the pilot nature of the study, no a priori sample size calculation was conducted. However, a post hoc power analysis based on the observed area under the ROC curve (AUC) indicated that the study achieved >80% power to detect a significant difference from a null AUC of 0.5 at a 5% significance level, confirming the reliability of the observed diagnostic accuracy.

#### 3.2.4. Sensitivity, Specificity, PPV, and NPV of SCORED-M

Diagnostic performance indicators, namely, sensitivity, specificity, PPV, and NPV, were calculated and compared across various cut-off scores to identify the most appropriate threshold for the optimal multivariate model. A score of 4 or above was determined to be the optimal threshold, yielding high sensitivity (0.97) and moderate specificity (0.46), with high NPV (0.97) and moderate PPV (0.43). Youden’s J statistic was 0.43. While Youden’s J statistic was marginally higher at a cut-off score of 5 or above (J = 0.50) compared to 4 or above (J = 0.43), the lower threshold was ultimately chosen due to its markedly superior sensitivity. At a score of 4, sensitivity reached 0.97, indicating that nearly all individuals with CKD were correctly identified. In contrast, sensitivity declined to 0.74 at the higher cut-off (Table 7). A comparison table of SCORED and SCORED-M is displayed in Table 8.

## 4. Discussion

### 4.1. Adjustment of Age Scoring Range

In the SCORED-M tool, the age-related scoring range was revised from 2–4 points to a narrower span of 1–3 points based on model comparison showing OR improvement from 1.48 to 1.70 and age-AUC enhancement from 0.61 to 0.62. This recalibration reflects a deliberate effort to moderate the influence of age as a standalone risk factor for CKD. While age remains a recognised predictor of CKD, excessive weighting may lead to an overestimation of risk, particularly among older adults who do not present with other clinical indicators. By reducing the maximum score allocated to age, the tool encourages a more balanced and multifactorial assessment of risk. This adjustment may also help reduce false-positive results in older populations, thereby improving specificity and overall diagnostic accuracy. Studies evaluating CKD-related quality of life and screening practices have highlighted the need for age-sensitive tools that do not disproportionately skew risk assessments [14,15].

### 4.2. Impact of Item Modifications in SCORED-M

The modification of individual items within the SCORED-M tool significantly enhanced its clinical relevance and diagnostic precision. A key revision involved replacing the item ‘*I have high blood pressure*’ with ‘*I have uncontrolled high blood pressure*’, thereby placing greater emphasis on poorly managed hypertension—a well-established and more critical risk factor for CKD progression [16]. This change is expected to improve the tool’s specificity by targeting individuals with a higher likelihood of disease advancement.

Similarly, multicollinearity analysis revealed a moderate positive association between the variables ‘*I am diabetic*’ and ‘*I have uncontrolled diabetes*’. This finding suggests that these variables are not independent and may convey overlapping information in the context of CKD risk modelling. Given this redundancy, the inclusion of both variables could inflate variance and reduce model interpretability. From a clinical and statistical standpoint, retaining the broader variable ‘*I am diabetic*’ is preferable for CKD screening. Diabetes, regardless of control status, is a well-established risk factor for CKD and routinely used in validated screening tools such as the SCORED and Kidney Failure Risk Equation models [2,17,18]. The KDIGO guideline likewise emphasises the importance of identifying diabetes as a primary driver of CKD progression, without necessarily stratifying by glycaemic control in initial screening contexts [2]. Therefore, simplifying the model by excluding ‘*I have uncontrolled diabetes*’ enhances parsimony while preserving predictive accuracy.

### 4.3. Justifications for Items Removed from SCORED

The item ‘*I have circulation disease in my legs*’ was excluded due to its comparatively poor performance among all predictors in the logistic regression model. This outcome suggests its limited discriminatory power in identifying individuals with CKD. While peripheral vascular disease is associated with systemic atherosclerosis, its direct predictive value for CKD is less robust compared to primary risk factors such as diabetes, hypertension, and albuminuria [2].

Multivariate analyses also led to the sequential exclusion of several other items due to limited incremental value in predicting CKD. These included (Q6) ‘*I have a history of heart attack or stroke*’, (Q3) ‘*I have or have had anaemia*’, (Q2) ‘*I am a woman*’, (Q8) ‘*I have circulation disease in my legs*’, and (Q13) ‘*I have a history of urinary problem(s)*’. Although cardiovascular disease and anaemia are common comorbidities of CKD, their presence may reflect disease progression rather than serving as early indicators [19]. Similarly, while sex and urinary symptoms may influence CKD presentation, they lack the specificity and predictive strength required for inclusion in a parsimonious screening model. The removal of the ‘female sex’ item reflects its lack of independent predictive value in this cohort, consistent with epidemiological patterns where females demonstrate lower CKD risk despite higher all-cause mortality. Future validation studies across diverse populations will examine sex interactions and consider sex-specific thresholds to ensure equitable performance across demographic groups.

The item ‘*I have circulation disease in my legs*’ was also excluded, due to its limited discriminatory power in identifying individuals with CKD. While peripheral vascular disease is associated with systemic atherosclerosis, its direct predictive value for CKD is less robust compared to primary risk factors such as diabetes, hypertension, and albuminuria The exclusion of these items aligns with the KDIGO recommendation to streamline screening tools by focusing on variables with high predictive utility and clinical relevance [2,19].

### 4.4. Justifications for Item Retention or Addition

Although the item ‘*I have a history of congestive heart failure or heart failure*’ yielded only marginal statistical significance (*p* = 0.059), it was retained in the optimal multivariate model due to its notably high OR (22.87), indicating a strong association with CKD. This decision is supported by evidence from Bang et al. [6], who identified congestive heart failure as a significant predictor of CKD in the original SCORED model. Heart failure contributes to renal hypoperfusion and neurohormonal activation, both of which accelerate kidney dysfunction [20]. Therefore, its inclusion enhances the clinical relevance of the model, especially in populations with cardiovascular comorbidities.

Additional variables retained in the optimal model include the self-reported items ‘*I have protein in my urine*’ (*p* < 0.005) and ‘*I have a history of renal disease*’ (*p* < 0.001), both of which demonstrated statistically significant associations with CKD. Proteinuria is a well-established marker of kidney damage and a key component in CKD staging and prognosis [21]. Similarly, a history of renal disease reflects prior structural or functional abnormalities, which are strong predictors of CKD progression [22]. These findings are consistent with the foundational analysis of the SCORED model, which emphasised the predictive strength of these variables in univariate and multivariate contexts.

### 4.5. Improvement of AUC in the SCORED-M

The comparative analysis of ROC curves revealed that SCORED-M demonstrated superior diagnostic performance over the original SCORED algorithm, with an AUC of 0.89 versus 0.79, respectively. This enhancement in AUC reflects the improved discriminative ability of SCORED-M to correctly classify individuals with and without CKD, thereby reinforcing its utility in population-level screening. These findings align with prior validation studies that emphasised the importance of model refinement to optimise sensitivity and specificity in CKD detection [6,7]. The improved accuracy of SCORED-M supports its adoption in clinical and community settings where early identification of CKD is critical to preventing progression and reducing associated cardiovascular risks [17].

### 4.6. Optimal Threshold Determination

The identification of a score of ≥4 as the optimal threshold for SCORED-M was based on a comparative evaluation of key diagnostic metrics, namely, sensitivity, specificity, PPV, and NPV. At this threshold, the tool demonstrated very high sensitivity (0.97) and high NPV (0.97), indicating a strong capability to identify individuals with CKD and rule out those without correctly. Meanwhile, it maintained moderate specificity (0.46) and PPV (0.43), helping reduce false-positive results while preserving screening efficiency. This balance between identifying accurate cases and minimising unnecessary follow-up supports the use of a score of ≥4 as a clinically practical and statistically robust threshold for early CKD detection [2,23]. Increasing the threshold to ≥5 may improve specificity (0.76), but this comes at the expense of reduced sensitivity (0.74), potentially leading to missed cases of early-stage CKD.

While Youden’s J statistic was marginally higher at a cut-off score of 5 or above (J = 0.50) compared to 4 or above (J = 0.43), the lower threshold was ultimately chosen due to its markedly superior sensitivity. In the context of screening, particularly for conditions like CKD where early detection is essential to prevent disease progression, sensitivity is often prioritised over specificity. Opting for a lower threshold helps minimise the risk of missing actual cases, even if it results in a greater number of false positives. This approach aligns with public health priorities, where the principal aim of screening is to maximise the identification of at-risk individuals, especially those who may be asymptomatic [24].

### 4.7. Resource-Saving Implications of the Optimal Threshold and Shortened Version

The KDIGO 2024 Guideline [4] emphasises the importance of early detection and risk stratification. Therefore, SCORED-M can be used for screening CKD, especially in high-risk populations such as individuals with diabetes, hypertension, heart failure, or those with a family history of CKD [2]. The application of a cut-off score of ≥4 in the SCORED-M tool for further verification of CKD status presents notable advantages in terms of resource efficiency. By focusing diagnostic efforts such as laboratory testing and clinical follow-up on individuals who meet or exceed this threshold, healthcare providers can reduce unnecessary investigations among those at lower risk of CKD. This targeted approach not only conserves essential resources, including clinical time, personnel, and diagnostic equipment, but also alleviates patient burden by avoiding unwarranted procedures and associated anxiety. It also enhances operational efficiency in screening programmes by streamlining case identification and prioritising care for individuals most likely to benefit from early intervention. According to the NKF [5], early and guideline-compliant screening for CKD improves detection rates and enhances institutional efficiency and care quality.

Utilising a shortened version of the CKD screening tool, such as SCORED-M, presents notable practical and clinical benefits. By condensing the questionnaire from nine to six items, SCORED-M improves accessibility and operational efficiency, particularly within high-demand environments like general practice and community health settings. The reduced length lessens the burden on respondents and enhances the likelihood of full completion, all while maintaining diagnostic integrity. Notably, SCORED-M has shown improved screening accuracy compared to the original version. The current study’s novelty stems from its tool optimisation and validation framework, providing an evidence base for scaling up to larger, multi-site, and multi-method studies. Although the study sample was modest, post hoc power estimation and effect size calculations support the statistical robustness of the main outcomes.

### 4.8. Comparison with Existing CKD Screening Tools

Unlike laboratory-dependent models such as the Kidney Failure Risk Equation (KFRE)—requiring eGFR, albuminuria, and serum analytes (AUC 0.80–0.88)—SCORED-M relies solely on self-reported risk factors, making it more feasible for primary care settings without immediate lab access [18,25]. Compared to the original SCORED (nine items, AUC 0.79), our modified six-item version demonstrates superior discrimination (AUC 0.89) while reducing respondent burden [6,11]. Other risk factor questionnaires show AUCs ranging from 0.57 to 0.88 but often require clinical data integration; SCORED-M’s fully self-administered design uniquely balances simplicity, accuracy, and accessibility for community screening programmes where resource constraints limit biomarker testing [26].

## 5. Limitations and Recommendations

This investigation offers an initial assessment of the modified SCORED-M screening tool for CKD using a pilot cohort. Although the results suggest encouraging gains in predictive accuracy and model simplicity, the relatively small sample size limits the extent to which these findings can be generalised. Smaller cohorts may fail to reflect the full spectrum of CKD risk factors across varied age groups, ethnic backgrounds, and coexisting health conditions, which could result in overfitting or exaggerated effect sizes. While participant characteristics align with typical CKD screening populations, the single-centre pilot design limits external validity, necessitating multi-site validation across diverse sociodemographic groups. Furthermore, limited statistical power may compromise the robustness of multivariate relationships and the dependability of threshold selection. To build a stronger evidence base, future research should aim to validate the SCORED-M tool in larger and more diverse populations, ideally through multicentre or longitudinal studies. Incorporating external validation samples and subgroup analyses will be vital to confirm the tool’s reliability, refine its scoring thresholds, and evaluate its applicability across a range of clinical and community contexts. While the modified SCORED-M tool focuses on self-reported variables to maximise its screening practicality, future large-scale validation studies should integrate objective clinical indicators, such as laboratory results and disease duration data, to improve predictive validity and fully capture the multifactorial nature of CKD risk. The findings of this pilot validation study highlight the potential of the SCORED-M tool as a pragmatic screening approach to support primary prevention and early detection of CKD in both clinical and community contexts. Future research expanding this tool’s validation with objective clinical indicators and longitudinal tracking could further strengthen its role in reducing CKD-related morbidity and healthcare costs.

A further limitation is that the CKD classification relied on a single eGFR measurement without repeat testing or systematic assessment of albuminuria, which may introduce classification bias and potential overestimation of CKD, as reported in previous studies comparing single versus repeated eGFR/ACR measurements. Consequently, the CKD status in this study should be interpreted as provisional, and future validation work will incorporate repeated biological measurements over time to confirm chronicity and reduce misclassification.

## 6. Conclusions

This pilot investigation provided an early evaluation of the modified SCORED screening tool for CKD (SCORED-M), highlighting that a simplified version comprising seven items can maintain clinical relevance while enhancing predictive accuracy. The refinement process involved removing items with minimal predictive contribution and incorporating variables more strongly linked to CKD risk, resulting in a model that better reflects current clinical insights. Despite the limited sample size, SCORED-M outperformed the original version, as evidenced by the higher values for AUC, sensitivity and NPV. A threshold score of ≥4 was identified as the most suitable cut-off, promoting efficient resource use in early CKD detection. Nonetheless, due to the small cohort, these findings should be interpreted with caution. Further research involving larger, multicentre cohorts is recommended to confirm the tool’s reliability, validate its performance across diverse populations, and optimise its use in clinical and community-based screening programmes.

## Figures and Tables

**Table 1:** Items in different versions of the screening tool for chronic kidney diseases.

Item No.		Scored (Bang 2007) [6]	SCORED-Rev	Full Model for Multivariate Analyses After Univariate Analysis	SCORED-M [Optimal Model]
1	Age				
	50–59	2	1	1	1
	60–69	3	2	2	2
	70 and above	4	3 [revised coding scheme]	3	3
2	I am a woman	1	1	1	---
3	I had or have anaemia	1	1	1	---
4	I have high blood pressure	1	--- [replaced by Q10]	---	---
5	I am diabetic	1	--- [replaced by Q11]	1	1
6	I have a history of heart attack or stroke	1	1	1	---
7	I have a history of congestive heart failure or heart failure	1	1	1	1
8	I have circulation disease in my legs	1	1	1	---
9	I have protein in my urine	1	1	1	1
10	I have uncontrolled high blood pressure		1 [replacing Q4]	1	1
11	I have uncontrolled diabetes		1 [replacing Q5]	---	---
12	I have a history of renal disease(s)		1 [newly added]	1	1
13	I have a history of urinary problem(s)		1 [newly added]	1	---
	Total items	9	11	11	6
	Max scores	12	13	13	8

**Table 2:** Parameters generated by univariate logistic regression.

		Univariate Analyses						
(1)	(2)	Item	*p*-Value	Odd Ratio	OR (C.I.)	Phi Coefficient (Unless Specified)	AUC	Included in the Full Model
✓		Age - 50–59 (2) - 60–69 (3) - ≥70 (4)	0.277	1.48	[0.73, 3.00]	0.189 (Kendall’s tau)	0.61	No
	✓	Revised Age - 50–59 (1) - 60–69 (2) - ≥70 (3)	0.161	1.70	[0.81, 3.59]	0.189 (Kendall’s tau)	0.62	Yes
✓	✓	I am a woman	0.473	0.74	[0.33, 1.68]	−0.067	0.47	Yes
✓	✓	I had or have anaemia	0.131	2.22	[0.79, 6.22]	0.142	0.56	Yes
✓		I have high blood pressure	0.050	2.70	[1.00, 7.24]	0.186	0.60	No
	✓	I have uncontrolled high blood pressure	0.01	2.95	[1.29, 6.75]	0.243	0.63	Yes
✓		I am diabetic	0.113	1.93	[0.86, 4.35]	0.148	0.58	Yes
	✓	I have uncontrolled diabetes	0.854	1.11	[0.36, 3.49]	0.017	0.51	No
✓	✓	I have a history of heart attack or stroke	0.096	2.32	[0.86, 6.27]	0.157	0.57	Yes
✓	✓	I have a history of congestive heart failure or heart failure	0.53	2.46	[0.15, 40.41]	0.06	0.51	Yes
✓	✓	I have circulation disease in my legs	0.621	0.80	[0.34, 1.91]	−0.046	0.48	Yes
✓	✓	I have protein in my urine	<0.001	5.97	[2.44, 14.64]	0.381	0.69	Yes
	✓	I have a history of renal disease(s)	<0.001	11.97	[4.38, 32.70]	0.498	0.77	Yes
	✓	I have a history of urinary problem(s)	0.071	0.43	[0.18, 1.08]	−0.17	0.41	Yes

(1) Original SCORED (9 items); (2) SCORED-rev (11 items).

**Table 3:** Model comparison using multivariate logistic regression after item removal.

		Model 1	Model 2	Model 3	Model 4	Model 5	Model 6
	SCORED (9 Items)	Complete Model (11 Items)	After Removing Q6 (10 Items)	After Removing Q6 and Q3 (9 Items)	After Removing Q6, Q3, and Q2 (8 Items)	After Removing Q6, Q3, Q2, and Q8 (7 Items)	After Removing Q6, Q3, Q2, Q8, and Q13 (6 Items) SCORED-M [Optimal Model]
Number of parameters (no. of independent variables + 1)	9 + 1 = 10	11 + 1 = 12	10 + 1 = 11	9 + 1 = 10	8 + 1 = 9	7 + 1 = 8	6 + 1 =7
−2 Log Likelihood	114.295	82.889	82.891	82.895	82.959	83.667	86.478
AIC value #	134.3	106.89	104.89	102.9	100.96	99.67	100.48
Nagelkerke R Square	0.287	0.557	0.557	0.557	0.556	0.551	0.529
Percentage of correctly predicted cases	76.7	84.5	84.5	84.5	82.8	85.3	83.6
Overall model quality	0.69	0.83	0.83	0.83	0.83	0.83	0.82
AUC	0.79	0.90	0.90	0.90	0.90	0.89	0.89

# Akaike Information Criterion (AIC) = −2 Log Likelihood + [2 × number of parameters]; Q2: I am a woman; Q3: I had or have anaemia; Q6: I have a history of heart attack or stroke; Q8: I have circulation disease in my legs; Q13: I have a history of urinary problem(s).

**Table 4:** Multivariate analyses using backward logistic regression (optimal model, six items, SCORED-M).

	Estimate (β)	OR (95% CI, Wald SE)	Wald	*p*-Value
(Intercept)	−6.160	0.002	14.449	<0.001
Age group (3 levels): 50–59 (1) 60–69 (2) 70 and above (3)	0.754	2.13	2.288	0.130
I am diabetic (1)	0.746	2.11	1.735	0.188
I have a history of congestive heart failure or heart failure	3.130	22.87	3.577	0.059
I have protein in my urine (1)	1.713	5.55	7.772	0.005 **
I have uncontrolled high blood pressure (1)	1.535	4.64	6.968	0.008 **
I have a history of renal disease (1)	2.866	17.57	19.748	<0.001 ***

** *p*-value < 0.01; *** *p*-value < 0.001.

**Table 5:** Item modifications and their justifications.

		Scored (Bang, 2007) [6]	Action	Scored-M (Optimal Model)	Justification
Scored	Q1	Age: I am between 50 and 59 years of age (score = 2) I am between 60 and 69 years of age (score = 3) I am 70 years old or older (score =4)	Modified	Age: I am between 50 and 59 years of age (score = 1) I am between 60 and 69 years of age (score = 2) I am 70 years old or older (score =3)	The original age group variable was replaced with a revised coding scheme, as it yielded a higher OR (1.70 vs. 1.48) and improved AUC (0.62 vs. 0.61).
	Q2	I am a woman	Removed	---	This item was excluded from the multivariate analyses due to its comparatively high *p*-value relative to the other variables.
	Q3	I had/have anaemia	Removed	---	This item was excluded from the multivariate analyses due to its comparatively high *p*-value relative to the other variables.
	Q4	I have high blood pressure	Replaced by Q10	See Q10	Multicollinearity was observed between these two variables (Q4 and Q10). Given this overlap, only one variable was retained for further analysis. “*I have uncontrolled high blood pressure*” was selected because it demonstrated stronger predictive performance, with a higher OR (2.95 vs. 2.70), greater AUC (0.63 vs. 0.60), and a more significant *p*-value (0.01) compared to the alternative. This item was retained in the optimal multivariate model due to its statistically significant *p*-value (0.008).
	Q5	I am diabetic	Retained	I am diabetic	Multicollinearity was identified between the variables (Q5 and Q11). Given this overlap, only one variable was retained for further analysis. “*I am diabetic*” was selected due to its stronger predictive performance, reflected in a higher OR (1.11 vs. 1.93) and greater AUC (0.51 vs. 0.58) compared to “*I have uncontrolled diabetes*”.
	Q6	I have a history of heart attack or stroke	Removed	---	This item was excluded from the multivariate analyses due to its comparatively high *p*-value relative to the other variables.
	Q7	I have a history of congestive heart failure or heart failure	Retained	I have a history of congestive heart failure or heart failure	Although the *p*-value for the item ‘*I have a history of congestive heart failure or heart failure*’ was only marginally statistically significant (*p* = 0.059), it was retained in the optimal multivariate model due to its notably high OR (22.87).
	Q8	I have circulation disease in my legs	Removed	---	This item was excluded from the multivariate analyses due to its comparatively high *p*-value relative to the other variables.
	Q9	I have protein in my urine	Retained	I have protein in my urine	This item was retained in the optimal multivariate model due to its statistically significant *p*-value < 0.005.
Potential modifications	Q10	I have uncontrolled high blood pressure	Replaced Q4	I have uncontrolled high blood pressure	See Q4
	Q11	I have uncontrolled diabetes	Not included	---	See Q5
	Q12	I have a history of renal disease(s). If yes, please specify:	Added	I have a history of renal disease(s). If yes, please specify:	This item was retained in the optimal multivariate model due to its statistically significant *p*-value (*p* < 0.001).
	Q13	I have a history of urinary problem(s). If yes, please specify:	Not included	---	This exclusion was justified by both empirical and methodological considerations. In the univariate logistic regression, the item yielded a weak predictive value (OR = 0.43), indicating limited utility in identifying CKD.

AUC: Area under the curve; CKD: Chronic kidney disease; OR: odds ratio.

**Table 6:** AUCs of SCORED and SCORED-M.

	AUC	z-Statistic	*p*-Value
Original SCORED	0.79	−2.45	0.014
SCORED-M6	0.89		

By DeLong’s test (implemented in the package “pROC” with function “roc.test()”).

**Table 7:** Sensitivity, specificity, PPV, and NPV of the SCORED-M6.

Threshold	Sensitivity	Specificity	PPV	NPV	Youden’s J
≥3	0.97	0.17	0.33	0.94	0.14
**≥4**	**0.97**	**0.46**	**0.43**	**0.97**	**0.43**
≥5	0.74	0.76	0.56	0.87	0.5
≥6	0.35	0.98	0.86	0.78	0.33
≥7	0.12	1.00	1.00	0.73	0.12

PPV: Positive predictive value; NPV: Negative predictive value.

**Table 8:** Comparison of Items and Scoring Between SCORED and SCORED-M.

SCORED (9 Items) [6]	SCORED-M (6 Items)
Age: 1. I am between 50 and 59 years of age (score = 2) 2. I am between 60 and 69 years of age (score = 3) 3. I am 70 years old or older (score = 4)	Age: 1. I am between 50 and 59 years of age (score = 1) 2. I am between 60 and 69 years of age (score = 2) 3. I am 70 years old or older (score = 3)
I am a woman (score = 1)	---
I had/have anaemia (score = 1)	---
I have high blood pressure (score = 1)	I have uncontrolled high blood pressure (score = 1)
I am diabetic (score = 1)	I am diabetic (score = 1)
I have a history of heart attack or stroke (score = 1)	---
I have a history of congestive heart failure or heart failure (score = 1)	I have a history of congestive heart failure or heart failure (score = 1)
I have circulation disease in my legs (score = 1)	---
I have protein in my urine (score = 1)	I have protein in my urine (score = 1)
---	I have a history of renal disease(s). If yes, please specify: (score = 1)
Maximum score: 12	Maximum score: 8

## Data Availability

The data presented in this study are available upon reasonable request from the corresponding author, as the data utilised in this study are confidential.

## Supplementary Materials

The following supporting information can be downloaded at: https://www.mdpi.com/article/10.3390/healthcare14050589/s1, Table S1: Standards for Reporting Diagnostic Accuracy Studies’ (STARD) 2015 guidelines.

## Abbreviations

AUC	Area under the curve
CI	Confidence interval
CKD	Chronic kidney disease
eGFR	Estimated glomerular filtration rate
ESKD	End-stage kidney disease
IHC	Integrated Health Clinic
OR	Odds ratio
ROC	Receiver operating characteristic
SCORED	SCreening for Occult REnal Disease
STARD	Standards for Reporting Diagnostic Accuracy Studies
TWC	Tung Wah College

## References

[1] Borges L.D., Dias H.H., Ferreira Ede S., Alves P.M., Silva B.O., Santos Kde P., Costa GDda Moreira T.R., Santos D.S., Cotta R.M.M. (2023). Prevalence of diabetes mellitus among individuals with chronic kidney disease: Systematic review and meta-analysis. J. Evid.-Based Healthc..

[2] Kidney Disease: Improving Global Outcomes (KDIGO). Clinical Practice Guideline for the Evaluation and Management of Chronic Kidney Disease. Kidney Int Suppl.b2024. https://kdigo.org/guidelines/ckd-evaluation-and-management/.

[3] Agvall B., Ashfaq A., Bjurström K., Etminani K., Friberg L., Lidẻn J., Lingman M. (2023). Characteristics, management, and outcomes in patients with CKD in a healthcare region in Sweden: A population-based observational study. BMJ Open.

[4] Levin A., Ahmed S., Carrero J.J., Foster B., Francis A., Hall R.K., Herrington W.G., Hill G., Inker L.A., Kazancıoğlu R. (2024). Executive summary of the KDIGO 2024 Clinical Practice Guideline for the Evaluation and Management of Chronic Kidney Disease: Known knowns and known unknowns. Kidney Int..

[5] National Kidney Foundation (NKF) (2025). CKD Data Analysis Strategy. https://www.kidney.org/sites/default/files/2025-03/622-9794_2503-ckd_dataanalysisprocess-3.17.2025-1.pdf.

[6] Bang H., Shoham D.A., Klemmer P.H., Falk R.J., Maxumdar M., Gipson D., Colindres R.E., Kshirsagar A.V. (2007). Screening for Occult Renal Disease (SCORED): A simple prediction model for chronic kidney disease. Arch. Intern. Med..

[7] Harward D.H., Bang H., Hu Y., Bomback A.S., Kshirsagar A.V. (2014). Evaluation of the SCORED questionnaire to identify individuals with Chronic Kidney Disease in a community-based screening program in rural North Carolina. J. Community Health Edu..

[8] Rovin B.J., Adler S.G., Barratt J., Bridoux F., Burdge K.A., Chan T.M., Cook H.T., Fervenza F.C., Gibson K.L., Glassock R.J. (2021). KDIGO 2021 Clinical Practice Guideline for the Management of Glomerular Diseases. Kidney Int. Suppl..

[9] National Kidney Foundation (2022). Estimated Glomerular Filtration Rate (eGFR). https://www.kidney.org/kidney-topics/estimated-glomerular-filtration-rate-egfr.

[10] CKD-EPI Equation, National Kidney Foundation’s Online Calculator. https://www.kidney.org/professionals/gfr_calculator.

[11] Bang H., Mazumdar M., Kern L.M., Shoham D.A., August P.A., Kshirsagar A.V. (2008). Validation and Comparison of a Novel Screening Guideline for Kidney Disease: KEEPing SCORED. JAMA Intern. Med..

[12] Schisterman E.F., Faraggi D., Reiser B., Hu J. (2008). Youden index and the optimal threshold for markers with mass at zero. Stat. Med..

[13] Bossuyt P.M., Reitsma J.B., Bruns D.E., Gatsonis C.A., Glasziou P.P., Irwig L., Lijmer J.G., Moher D., Rennie D., de Vet H.C. (2015). STARD 2015: An updated list of essential items for reporting diagnostic accuracy studies. Radiology.

[14] Flythe J.E., Karlsson N., Sundgren A., Cordero P., Grandinetti A., Cremisi H., Rydén A. (2021). Development of a preliminary conceptual model of the patient experience of chronic kidney disease: A targeted literature review and analysis. BMC Nephrol..

[15] Hays R.D., Kallich J.D., Mapes D.L., Coons S.J., Carter W.B. (1994). Development of the kidney disease quality of life (KDQOL) instrument. Qual. Life Res..

[16] Ku E., Lee B.J., Wei J., Weir M.R. (2019). Hypertension in CKD: Core Curriculum 2019. Am. J. Kidney Dis..

[17] Islam F., Siddiqui H.J., Khalid A., Farrukh G., Yousaf S., Ahmed A. (2023). Chronic kidney disease and associated risk factors among patients with type-2 diabetes mellitus in a tertiary care hospital. Pak. Armed Forces Med. J..

[18] Tangri N., Grams M.E., Levey A., Coresh J., Appel L.J., Astor B.C., Chodick G., Collins A.J., Djurdjev O., Elley C.R. (2016). Multinational assessment of accuracy of equations for predicting risk of kidney failure: A meta-analysis. JAMA.

[19] Sarnak K.J., Amann K., Bangalore S., Cavalcante J.L., Charytan D.M., Craig J.C., Gill J.S., Hlatky M.A., Jardine A.G., Landmesser U. (2019). Chronic kidney disease and coronary artery disease. J. Am. Coll. Cardiol..

[20] Shahzad M., Ahmed S., Ahsan M., Sulfiqar E., Hurjkaliani S., Thakur T., Khan R., Sethi P., Daoud M., Goyal A. (2025). Trends and disparities in heart failure mortality with and without chronic kidney disease in a nationwide retrospective analysis. Sci. Rep..

[21] Levey A.S., Eckardt K.U., Tsukamoto Y., Levin A., Coresh J., Rossert J., Zeeuw D.D.E., Hostetter T.H., Lameire N., Eknoyan G. (2005). Definition and classification of chronic kidney disease: A position statement from Kidney Disease: Improving Global Outcomes (KDIGO). *Kidney Int*..

[22] Mutter S., Valo E., Aittomầki V., Nybo K., Raivonen L., Thorn L.M., Forsblom C., Sandholm N., Würtz P., Groop P.-H. (2022). Urinary metabolite profiling and risk of progression of diabetic nephropathy in 2670 individuals with type 1 diabetes. Diabetologia.

[23] Nemecek B.D., St Peter W.L., Hong L.T., Anderson E.R., El Nedidy W.S. (2025). Current practices in estimating kidney function: Insights from a cross-sectional survey. JACC J. Am. Coll. Clin. Pharm..

[24] World Health Organization (2020). Screening Programmes: A Short Guide. Increase Effectiveness, Maximize Benefits and Minimize Harm. WHO Regional Office for Europe. https://apps.who.int/iris/bitstream/handle/10665/330829/9789289054782-eng.pdf.

[25] Tangri N., Stevens L.A., Griffith J., Tighiouart H., Djurdjev O., Naimark D., Levin A., Levey A.S. (2011). A predictive model for progression of chronic kidney disease to kidney failure. JAMA.

[26] Collins G.S., Omar O., Shanyinde M., Yu L.M. (2013). A systematic review finds prediction models for chronic kidney disease were poorly reported and often developed using inappropriate methods. J. Clin. Epidemiol..

